# Recent Progress in Resonant Acoustic Metasurfaces

**DOI:** 10.3390/ma16217044

**Published:** 2023-11-05

**Authors:** Dongan Liu, Limei Hao, Weiren Zhu, Xiao Yang, Xiaole Yan, Chen Guan, You Xie, Shaofang Pang, Zhi Chen

**Affiliations:** 1 College of Science, Xi’an University of Science and Technology, Xi’an 710054, China16608207879@163.com (C.G.);; 2 Department of Electronic Engineering, Shanghai Jiao Tong University, Shanghai 200240, China; 3 Department of Applied Physics, Northwestern Polytechnical University, Xi’an 710129, China; c2002z@nwpu.edu.cn

**Keywords:** acoustic wave, acoustic metasurfaces, reflection, transmission, absorptive, tunability, wavefront manipulation

## Abstract

Acoustic metasurfaces, as two-dimensional acoustic metamaterials, are a current research topic for their sub-wavelength thickness and excellent acoustic wave manipulation. They hold significant promise in noise reduction and isolation, cloaking, camouflage, acoustic imaging, and focusing. Resonant structural units are utilized to construct acoustic metasurfaces with the unique advantage of controlling large wavelengths within a small size. In this paper, the recent research progresses of the resonant metasurfaces are reviewed, covering the design mechanisms and advances of structural units, the classification and application of the resonant metasurfaces, and the tunable metasurfaces. Finally, research interest in this field is predicted in future.

## 1. Introduction

The efficient manipulation of electromagnetic or acoustic waves is a prominent area of natural sciences. The metasurface provides a new idea for wave manipulation. In 2011, Yu et al. proposed the theory of interfacial phase discontinuity [1]. “V”-shaped microstructures can be designed in sub-wavelength materials based on the theory, and these materials, known as metasurfaces, can be controlled by geometric parameters of the structure to obtain the phase change of 0 to 2π [2], and consequently, arbitrarily regulate electromagnetic wave propagation, driving a boom in electromagnetic metasurfaces [3,4,5,6]. An electromagnetic metasurface has the advantage of a strong modulation, thin size and various production capabilities. Similar to electromagnetic waves, the concept of electromagnetic metasurfaces was quickly extended to the acoustics field. Acoustic metasurfaces can also achieve an arbitrary modulation of acoustic wave propagation. Li et al. designed a two-dimensional ultrathin acoustic metasurface with a space-coiling structure and realized arbitrary regulation of the reflected acoustic wave both theoretically and experimentally [7,8].

Space-coiling structures [9] and resonance structures are the main two types of structural units for building acoustic metasurfaces. The space-coiling structure achieves relative control of the phase shift by accumulating travel distances of acoustic waves in the coil channel. Furthermore, the resonant structure has the advantage of manipulating large wavelengths with a smaller structure, and the acoustic metasurface constructed by the resonant unit realizes anomalous reflection and focusing at deep subwavelengths. In addition, efficiency is an important issue in the design of acoustic metasurfaces. For example, perfect absorbers and bianisotropic metasurfaces were used in perfect anomalous reflection and transmission. However, it is worth noting that the functionality of these metasurfaces is fixed and they operate only at a single operating frequency or a narrow frequency range. Therefore, the design of tunable acoustic metasurfaces has become a fascinating topic. Such metasurfaces should be tuned either by the geometrical parameters of the structure unit or by external physical fields (e.g., electromagnetic or force fields).

Here, we review the recent research progresses of the resonant metasurfaces. This paper is structured as follows. Section 2 presents the resonance mechanism and development of the structure unit for resonant metasurfaces. Section 3 surveys three main types of the metasurface and the representative phenomena and applications, including acoustic cloaking, sound absorption, acoustic focusing and so on. Section 4 summarizes the classification and development of tunable metasurfaces. Some main challenges and future outlooks towards developing resonance metasurfaces are given in Section 5. The detailed principles of metasurfaces can be found in References [10,11].

## 2. The Resonance Structure Units

The construction of structural units is crucial in developing acoustic metasurfaces. These units must fulfill the necessary requirements, including the 2π phase change and being as small as possible. The resonant structure unit that controls large wavelengths with a small size precisely meets this requirement, and it is increasingly researched. These resonant units (e.g., Helmholtz resonance, thin film resonance) can induce unipolar or dipole resonance in the entire structure through various resonance mechanisms and can achieve negative effective modulus or mass density, which is a benefit for adjusting parameters such as phase and resonant frequency. The following provides a concise overview of the resonance principle and research progress on Helmholtz resonance and thin film resonance.

### 2.1. Helmholtz Resonance Unit

The Helmholtz resonator (HR) is a basic acoustic resonance system that features a cavity surrounded by a rigid wall and an elongated neck. According to the acoustic force analogy theory, this system can be analogized as a spring-mass system, where the cavity’s neck is viewed as a mass and the cavity as a spring. Near the resonant frequency, the incident sound wave resonates strongly in HR and the body cavity gathers a large amount of energy, causing strong vibration of the acoustic medium at the neck. The vibration intensity is much greater than the excitation intensity of incident sound waves, and the dynamic response of the material is not synchronized with the excitation of external sound waves, exhibiting opposite response patterns. That is, when external sound waves compress the medium, the acoustic medium in the material undergoes an expansion motion. When sound waves stretch the medium, it undergoes compression. Therefore, a negative dynamic response occurs and the dynamic elastic modulus of the material is negative near the resonant frequency [12,13,14,15,16,17,18].

**Figure 1:**
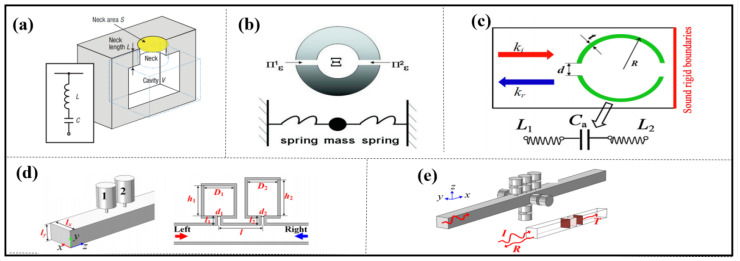
The HR structures used for acoustic wave manipulation. (**a**) schematic cross-sectional view of a Helmholtz resonator, reproduced from [12]; (**b**) a model of double ‘c’ resonator (DCR), reproduced from [21]; (**c**) cross-sectional diagram of one double-split hollow sphere (DSHS), reproduced from [19]; (**d**) three-dimensional (3D) view and cross-section of the system of the asymmetric acoustic wave guide with shunted HRs, reproduced from [22]; (**e**) 3D views of the absorber, reproduced from [23].

HRs offer several benefits including a straightforward design, ease of assembly, and a lengthy lifespan. Depending on their structural features, these resonators can be classified into three categories: HR, HR array, and HR-like units. Based on the physical properties of HRs, ultrasonic metamaterials were proposed by Fang in 2006, as shown in Figure 1a, consisting of an array of subwavelength HRs with designed acoustic inductance and capacitance. These materials have an effective dynamic modulus with negative values near the resonance frequency and offer the possibility of realizing applications such as acoustic negative refraction [12]. Similarly, the following structures [19,20,21], some shown in Figure 1b,c, again realize negative effects in specific frequency bands.

Long et al. present the mechanism for the asymmetric absorption of acoustic waves in a two-port transparent waveguide system by shunting detuned HR pairs in cascade, as shown in Figure 1d. Acoustic absorption in multiple bands or broadbands is attained by placing several HRs within a waveguide. This design advances the concept of asymmetric acoustic manipulation in passive two-port systems (see Figure 1e) [22,23].

An HR-like unit is constructed by inserting one or more separating plates with a small hole into the interior of an HR. The multi-order sound absorption mechanism can be achieved so that with the original absorption peak and the structural size unchanged, multiple near-perfect peaks are obtained in higher frequencies by a perforated composite Helmholtz resonator (PCHR) unit [24]. This work offers a new guidance for the achievement of a wider absorption band and has great potential in engineering applications.

### 2.2. Membrane Resonance Unit

Thin-film acoustic metamaterials can exhibit negative mass and bulk modulus, as well as double negativity within specific frequency ranges. The thin-film unit can also be analogized as a spring-mass system [25], where the mass block is viewed as the mass model and the preloading of the thin film as a spring. At the non-resonant frequencies, the thin film, restricted by the acoustic wave and the mass, vibrates near the equilibrium position; that is, all the components move simultaneously, and then the effective and static mass densities become equal. At the resonant frequencies, the cavity accumulates a considerable amount of energy. This energy hinders the synchronized motion of the thin-film structure and the phase reversal of the inner mass and spring occur. When the inner mass momentum exceeds that of the outer mass, the loading force and response acceleration are in the opposite direction, resulting in a negative effective mass density.

A double-layer thin-film structural unit is constructed by replacing the lower hard boundary with a thin film. This structure exhibits two dipolar modes that are comparable to those of a single thin film unit; hence, the feature of negative effective mass density is mostly retained. In addition, a new resonance mode has also emerged in the double-layer thin-film structure, and the relative vibration of compression/expansion occurs between the two membranes while the center of mass remains stationary, resulting in a negative effective bulk modulus [25,26,27,28].

**Figure 2:**
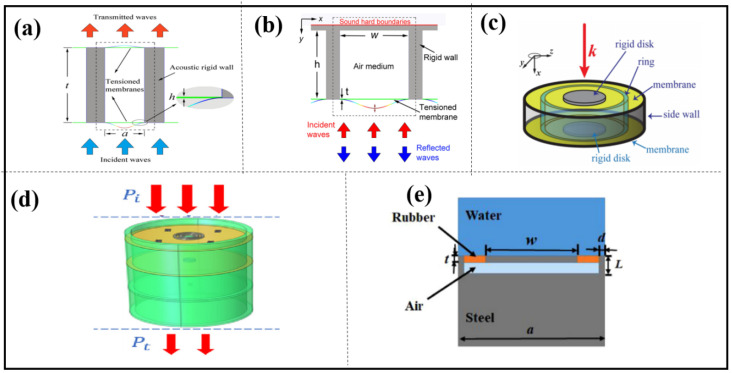
The membrane resonant structures used for acoustic wave manipulation. (**a**) Schematic drawing of subunit for the proposed reflection metasurface, reproduced from [40]; (**b**) schematic drawing of transmission metasurface consisting of a series of structurally simple microunits, reproduced from [49]; (**c**) schematic drawing of the double-negative metamaterial, reproduced from [29]; (**d**) schematic of the sandwich structure of double-membrane-type acoustic metamaterials combined with a Helmholtz resonator, reproduced from [30]; (**e**) metasurface unit cell constructed by steel, reproduced from [31].

Compared to HRs, a membrane structure unit (Figure 2a,b) is very sensitive, and the tension is difficult to control and maintain for a long time and may change sharply over time or change slightly with temperature and humidity. We can categorize them into three groups: thin films, thin plates and thin-film-like structures.

Yang et al. presented a structurally and conceptually simple double-negative acoustic metamaterial comprising two coupled membranes. Owing to its symmetry, the system can generate both monopolar and dipolar resonances that are separately tunable, thereby making broadband double negativity possible, as shown in Figure 2c [29]. A sandwich structure of double-membrane-type acoustic metamaterials combined with a Helmholtz resonator, as shown in Figure 2d, was designed by Li et al. in 2023, which is presented with both a pleasant mechanical nature and admirable acoustic insulation at a low frequency [30].

The design of the thin plate unit is illustrated in Figure 2e. It comprises a steel plate of width *w* connected to two steel supports using rubber spacers that hold the plate above an air cavity which creates an impedance mismatch that is used to maximize the reflected energy. The unit functions when hit by an incident wave, in turn causing the plate to vibrate. Similar to the mechanism seen in the membrane-type unit, the unit vibration causes a wave phase shift in the water and different reflected phases can be obtained [31].

Li et al. proposed a membrane-like unit consists of rotatable anisotropic three-component resonators which can induce non-degenerate dipolar resonance, causing an evident phase change in low frequencies. Compared with the monopole resonance widely used in HRs, the polarization direction of the dipole resonance is a new degree of freedom for phase manipulation. The phase profile can continuously change by rotating the anisotropic resonators [32].

In addition, researchers have investigated numerous resonance units, establishing a strong basis for the development of metasurfaces [7,19,20,21,30,33,34,35,36,37,38,39,40,41,42,43,44,45,46,47,48].

## 3. Acoustic Metasurface

An acoustic metasurface is an artificial two-dimensional acoustic metamaterial [50] with a thickness less than the wavelength; they have a broad range of applications and are employed in anomalous reflection, transmission, focusing, absorption, cloaking and other fields. The resonant acoustic metasurface is mainly composed of the Helmholtz or thin-film resonant structures mentioned in Section 2. In this section, acoustic metasurfaces are reviewed, including reflection, transmission and absorption metasurfaces.

### 3.1. Reflection Acoustic Metasurface

Acoustic metasurfaces constructed with Helmholtz resonators (HR) have successively achieved exotic acoustic phenomena, such as anomalous reflections, carpet cloak, focusing lens, acoustic diffusion, etc., by tuning structural parameters such as split-hole diameters, the spatial distance of the units and the volume of the cavities [19,20,51,52,53,54,55,56,57].

**Figure 3:**
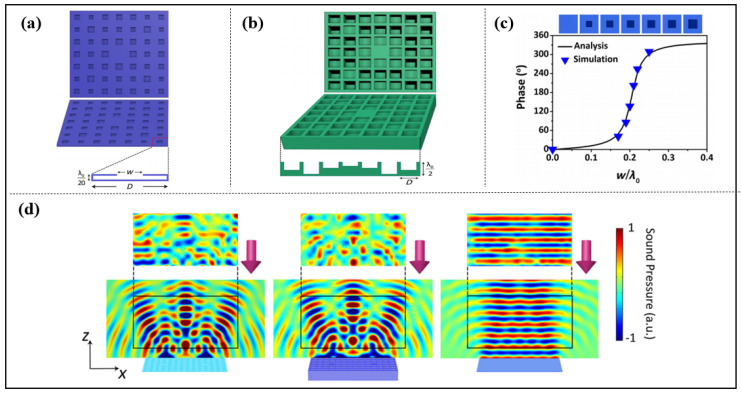
(**a**) The proposed metasurface-based Schroeder diffuser (MSD); (**b**) a two-dimensional Schroeder diffuser (2D SD); (**c**) the analytical and simulated relationship between the phase shift and the geometrical parameter w of the MSD. The triangles represent the discrete points for generating the phase of 0–2π × 6/7 with a step of 2π × 1/7; and (**d**) the measured (**upper**) and simulated (**lower**) scattered acoustic field distributions of the MSD, SD and flat plate in the *x*-*z* plane. Reproduced from [58].

With the concept of phase modulation of the acoustic metasurface’s structure, Zhu et al. proposed an ultrathin metasurface-based Schröder diffuser, which is similar to a Helmholtz resonator. The reflection phase can be varied from 0 to 2π by adjusting the width of the aperture *w*. The composition of the Schröder diffuser metasurface achieves a relatively efficient acoustic diffuse reflection, as shown in Figure 3 [58].

**Figure 4:**
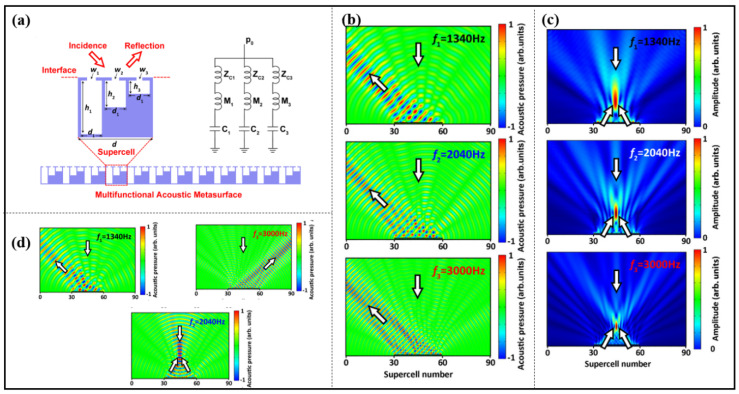
(**a**) (**Left**) 2D schematic diagram of the supercell of a multifunctional acoustic metasurface. A supercell consists of three cavities with different depths. (**Right**) the effective circuit model of the proposed supercell of the metasurface. (**b**) The simulated results for achromatic −45° extraordinary reflection at the three frequencies. (**c**) The corresponding results for achromatic acoustic focusing at (0 m, 0.6 m). (**d**) The simulated and experimental acoustic pressure distributions of the extraordinary reflection and acoustic focusing at the three frequencies. Reproduced from [59].

In order to further broaden the application of metasurfaces, researchers have proposed the concept of multi-band metasurfaces, as shown in Figure 4a, which achieve anomalous reflection, focusing and diffusing in multiple frequency bands by connecting different HRs with different resonant frequencies in parallel, as shown in Figure 4 [59,60,61].

**Figure 5:**
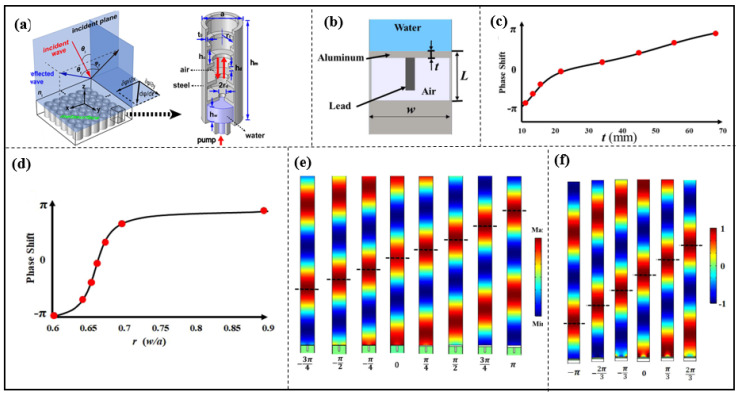
(**a**) Schematic of a square lattice of circular holes with gradient annular bumps; reproduced from [62]; (**b**) Metasurface unit cell constructed by aluminum; (**c**) phase shift as a function of ratio *r* for ali unit; (**d**) phase shift as a function of ratio *r* for steel unit; (**e**) phase shift as a function of thickness *t* for aluminum; (**f**) phase shift as a function of thickness *t* for steel; reproduced from [31,72].

In addition to bandwidth, efficiency is also one of the factors to be taken into account in designing metasurfaces. Li et al. proposed an acoustic metasurface which was constructed by a square lattice of circular holes with gradient annular bumps (see Figure 5a). The numerical results show that the wavefront of the reflected wave can be manipulated over a wide frequency range and the gradient unit cells can suppress the parasitic reflection [62].

The parameter optimization method, based on a genetic algorithm, was applied by Zhou et al. to construct a passive acoustic metasurface with stack-up HR units. Ultrabroadband and wide-angle carpet cloaking was realized [63].

Zhou et al. proposed an HR-like unit by designing a reflection metasurface for underwater sound steering, of which the thickness is tens times less than the wavelength. It was demonstrated that the local design based on the Generalized Snell Laws (GSL) does not work well, especially for a large reflection angle. The nonlocal design via the lattice diffraction theory (LDT), which was implemented using an optimization method, can obtain wavefront modulation with high efficiency [64].

Based on a topology optimization method, the optimized microstructure elements were designed by weakening vibration coupling between neighboring units, and precise wavefront manipulation including anomalous reflection with a steep angle, conversion from a propagating mode to an evanescent mode and near-field focusing with super-resolution were demonstrated by Zhou et al. in 2022 [64,65].

Thin-film structural units are also receiving attention because of their ultrathin property. Phase changes of 2π are achieved by adjusting the parameters of the structural units, such as width, thickness, Young’s modulus and mass density of the unit material, and the acoustics metasurfaces (AMS) can obtain acoustic phenomena, like anomalous reflections and focusing, and acoustic cloak [49,66,67,68,69].

In addition, acoustic phenomena such as anomalous reflections and reflection focusing can also be achieved by applying the film attached with different masses to adjust the tension or by choosing a film with masses of different sizes [70,71].

Chen et al. proposed a membrane-type unit which consists of an aluminum [72] (see Figure 5b,c,e) or steel [31] box (see Figure 2e and Figure 5d,f) with an air cavity and a lead mass attached to the top inside. Extremely thin metasurfaces with this resonant unit (λ/61.7) [31] were constructed to demonstrate anomalous reflection, sharp focusing, self-bending and carpet cloaking for waterborne sound [31,72].

**Figure 6:**
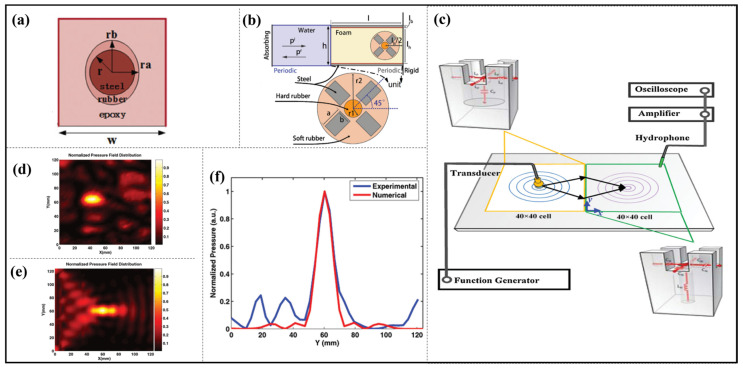
(**a**) Schematic of the proposed anisotropic resonant unit; reproduced from [73]. (**b**) The calculation region and a magnified view of the geometry of the inclusion; reproduced from [74]. (**c**) Schematic of the experimental setup. The sample with positive index and negative index (PI-NI) interface is composed of an array of the designed different HRs from an aluminum plate. Unit cells of each half part and the corresponding inductor–capacitor circuit analogy are shown in the insets; the normalized pressure field distribution at 60.5 kHz, (**d**) measured, and (**e**) simulated pressure field map of the acoustic NI metamaterial and (**f**) line plot of pressure field crosses the focal plane parallel to interface. Reproduced from [78].

A thin-film-like resonant structure which consists of epoxy resin and an elliptical rubber-coated steel core, as shown in Figure 6a, is presented, and the phase is controlled by adjusting the radius of the steel core, and the acoustic metasurface with this unit is further designed to achieve acoustic phenomena such as waterborne acoustic anomalous reflections, planar acoustic lenses and acoustic cloaks [73].

In addition, a multiple--resonant unit, based on rectangular foam, was bonded between two steel sheets and a multimass inclusion that was composed of a hard-rubber cylinder surrounded by four rectangular steel rods and embedded in a soft-rubber cylinder (see in Figure 6b). Its resonance effect was induced by changing the radius of the soft-rubber cylinder or rotating the angle of the multimass, and so the reflected wavefront achieved waterborne anomalous reflections, wide-angle broadband focusing and acoustic cloak [32,74,75].

Moreover, a metasurface with tube resonators instead of HRs which realized full-angle reflection was proposed by Liu et al. [76]. A deep subwavelength acoustic reflection metasurface (<λ/16) with meta-molecules was combined with two structural units; that is, hollow tubes and split-hole hollow spheres. The metasurface achieved abnormal reflection with 800 Hz bandwidth [77].

### 3.2. Transmission Acoustic Metasurface

Due to the unique properties of reflection metasurfaces, strong energy transfer can be achieved by using only hard boundary conditions during the design process. However, a high transmission efficiency and a 2π change in transmission phase are needed to simultaneously satisfy transmission metasurfaces. Furthermore, the insufficient energy transmission results in significant energy dissipation and loss, and the designed acoustical devices in this manner are highly inefficient, which greatly hinders the application of metasurfaces.

Zhang et al. in 2009 firstly demonstrated an ultrasound wave focusing through a flat acoustic metamaterial lens in an experiment, and the metasurface was composed of a planar network of subwavelength HRs (see in Figure 6c–f). They observed a tight focus half-wavelength in width at 60.5 kHz by imaging a point source and a variable focal length at different frequencies. This result was in excellent agreement with a numerical simulation from a transmission line model in which the effective mass density and compressibility were derived [78].

**Figure 7:**
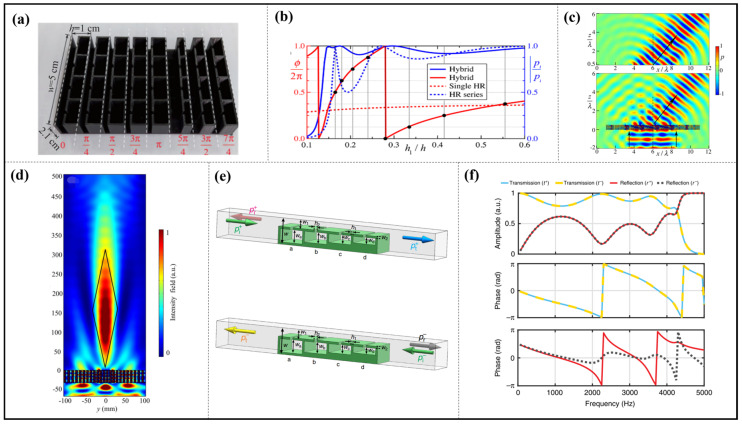
(**a**) An array of passive elements, (**b**) phase shift (red, solid) and transmission rate (blue, solid) of the hybrid structure as a function of height ratio h_1_ = h (or, correspondingly, h_3_ = h), and a comparison with that of HRs (red and blue dashed), reproduced from [34]; (**c**) anomalous refractions of theoretical (**up**) and simulated (**down**) pressure fields, reproduced from [80]; and (**d**) acoustic metasurface for the non-diffracting Bessel beam, reproduced from [79]. Study of a bianisotropic acoustic cell. (**e**) Geometry of a cell with four side-loaded resonators. The height of the HRs is varied to create different bianisotropic responses. Definition of the forward (+) and backward (−) illuminations; (**f**) amplitude and phase of the transmission and reflection coefficients of an arbitrary cell. Reproduced from [83].

A hybrid unit consisting of deep subwavelength cavities, which are a series connection of HRs, and a straight pipe at the open side of the HRs are used to construct the transmission screen, as shown in Figure 7a. A series connection of HRs acts as lumped elements, achieving the phase shift of the incident acoustic field; the straight pipe effectively matches the acoustic impedance based on the Fabry–Perot resonance, effectively enhancing the sound transmission (see Figure 7b). The theoretical and numerical results demonstrate that some excellent wavefront manipulations with anomalous refraction (see Figure 7c), non-diffracting Bessel beam (see Figure 7d), self-bending beam, focusing, effective tunable acoustic negative refraction, three-dimensional acoustic collimated self-accelerating beam, engineering acoustic beams, and switching on/off acoustic energy flow are obtained, as partly shown in Figure 7c,d [34,79,80,81,82].

Li et al. proposed and experimentally verified the bianisotropic unit, minimizing the losses (see Figure 7e,f). Three refractive metasurfaces based on the bianisotropic unit can redirect a normal incident plane wave to 60°, 70° and 80° in the transmission direction, and their efficiency is over 90%, which is much higher than the corresponding generalized Snell’s law-based designs (81%, 58% and 35%) [83]. An approach in which the power flow conformal design methodology and bianisotropic units were combined was proposed by Peng et al. As a demonstration in Reference [84], a transmission-type acoustic metasurface with maximum power efficiency was designed to focus sound plane waves in the near field at 3000 Hz from aspects of numerical simulations and experiments.

**Figure 8:**
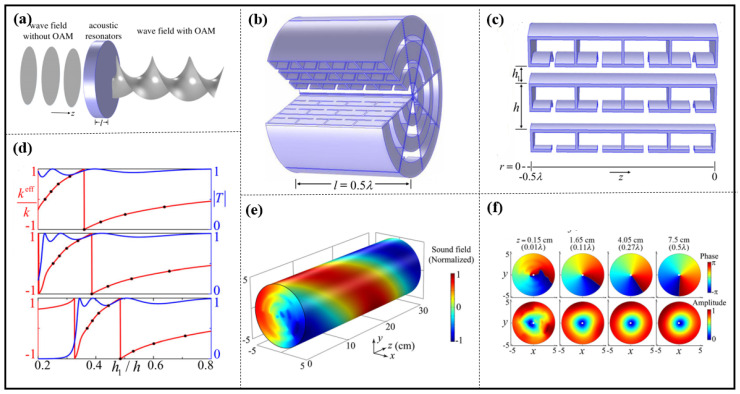
(**a**) Illustration of a resonant planar layer (blue) converting an in-coming axisymmetric wave without orbital angular momentum (OAM) to an outgoing beam with a helical wave front-carrying OAM (wave fronts are shown in gray), (**b**) schematic of the assembled layer consisting of eight fanlike sections of resonators, (**c**) an individual section consisting of three rows of resonators in the radial r direction, (**d**) the effective wave number *k^eff^* (red; normalized by *k* = 2π/*λ*) and transmission coefficient [T] (blue), (**e**) airborne sound pressure field on the outgoing surface of the planar layer, (**f**) phase (**top**) and amplitude (**bottom**) of the field at four cross-sections, illustrating the transition from the near to the far field, where the geometric centers of the cross-sections are denoted by the white dots. Reproduced from [85].

Jiang et al. constructed the planar layer as an assembly of eight fanlike sections of resonators over the whole azimuth (see Figure 8b). Each individual section was configured to be composed of three rows of resonators in the radius (more rows can be employed for a larger radius). Each row consisted of four lumped Helmholtz cavities and a straight pipe that could flexibly manipulate phases (or wave numbers *k^eff^*), as shown in Figure 8c. The combination of cavities and pipes provides hybrid resonances that overcome the impedance mismatch between the resonators and the surrounding air for a high transmission (see Figure 8d). They use the resonances in a planar layer of half-wavelength thickness to twist wave vectors of an in-coming plane wave into a spiral phase dislocation of an outgoing vortex beam with orbital angular momentum (OAM) (see Figure 8a,e,f). Their acoustic resonance-based OAM production via manipulating effective wave numbers, *k^eff^*, bears the advantages of high efficiency, compact size and planar profile [85].

**Figure 9:**
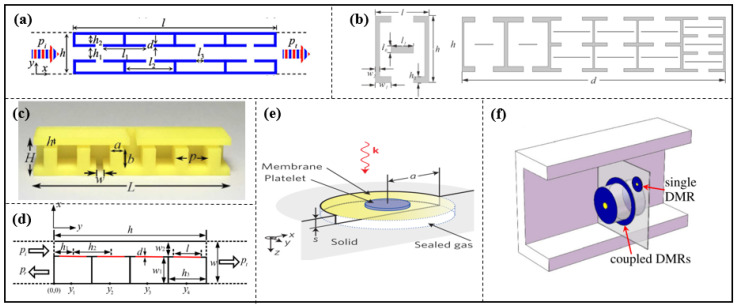
(**a**) Schematic of the symmetric unit, reproduced from [86]; (**b**) the top view of the first subunit. In order to conveniently calibrate the parameters of the structure, the figure is not the actual scale (**left**) schematic demonstration of the designed sample consisting of six subunits (**right**), reproduced from [87]; (**c**) HR unit with rectangular ridges inside, reproduced from [89]; (**d**) schematic illustration of an individual element of the metasurface made of four decorated membrane resonators and a straight pipe. Red solid lines refer to membranes, reproduced from [91]; (**e**) schematic illustration of the unit cell’s components and geometry. Here, a is the radius of the membrane, s is the depth of the sealed gas cell and k denotes the incident wavevector, reproduced from [92]; and (**f**) schematic cutoff view of the flat panel composite absorber. Reproduced from [93].

In addition, structural units, such as a symmetric unit coupling two layers of four HRs with a straight pipe [86], a single row of HRs with varying geometric parameters [87], dumbbell-shaped double-split hollow spheres (DSDSHS) [88], HRs with rectangular ridges inside [89], and a design approach of passive and reciprocal [90], have been proposed to enable flexible manipulation of transmitted acoustic waves (see Figure 9a–c) [86,87,88,89,90].

Compared to Helmholtz-types, the membrane-type is helpful to design a metasurface with smaller dimensions. A membrane-type unit, which consists of a cavity filled with air and two elastic membranes on the ends of cavity, is designed by Zhai et al. (see Figure 2a). By appropriately changing the thickness of the membranes to modulate the phase, the steering of the transmitted wave trajectory is demonstrated and some extraordinary phenomena are realized at 3.5 kHz, such as planar acoustic axicon, acoustic lens, the conversion from spherical waves to plane waves, and the transformation from propagating waves to surface waves [40].

A membrane-type hybrid unit with four HRs connecting to a straight pipe was presented to construct metasurfaces by Lan et al. Each resonator is an air-filled cavity with a rigid back and sealed with a membrane. The structure is similar to the HR unit [34,79,80,81,85], and the membrane corresponds to the short neck of the HR [91] (see Figure 9d). It is demonstrated that high transmission efficiencies, acoustic phenomena such as anomalous refraction, cloak based on flat focusing, self-bending beams, conversion of propagating waves to surface waves and negative refraction can be realized.

### 3.3. Absorption Acoustic Metasurface

Noise has become a problem topic in recent years as excessive noise not only affects people’s daily work and study but also impacts the performance, precision, reliability and safety of modern equipment. Traditional absorptive acoustic materials, such as acoustic sponge, multi-hollow fiber materials, etc., can only exhibit excellent performance in the high-frequency band, but relatively poor performance in the low-frequency band. According to acoustic theory, it is necessary to ensure that the size of the noise reduction material and the wavelength of the low-frequency noise are within an order of magnitude; that is, the thickness of the absorptive material must be used in the scale of decimeter or meter. So, the design of absorptive acoustic materials at low-frequency ranges is a very challenging and urgent issue.

An impedance-matched surface by using membrane units, as demonstrated by Ma et al. (see Figure 9e), can generate hybrid resonances due to multiple reflections between the membrane and reflective hard wall, and it can completely absorbed in one or multiple frequencies [92]. Using subwavelength decorated membrane resonators (DMRs) as basic units (see Figure 9f), Yang et al. experimentally achieved perfect sound absorption, up to 99.7%, even at a large airborne wavelength of up to 1.2 m [93]. It is demonstrated that the maximum absorption with a back-reflecting surface from two-sided incidence can reach 100%, and it was attained by the hybridized resonances [94].

**Figure 10:**
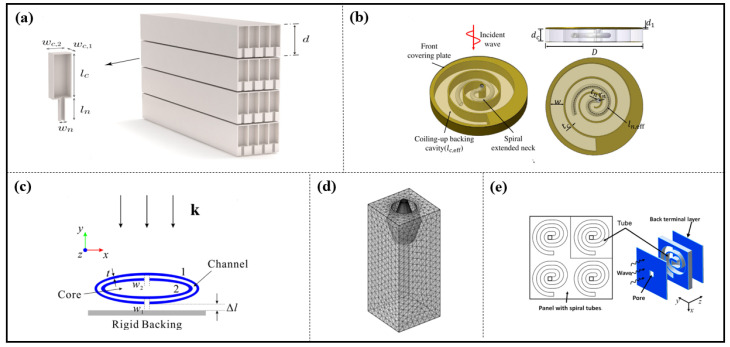
(**a**) Conceptual view of the thin panel placed on a rigid wall with N = 4 layers of square cross-section HRs, reproduced from [95]; (**b**) illustration of a resonator with a spiral extended neck and a coiled backing cavity, reproduced from [97]; (**c**) cross-sectional schematic of the absorber composed of two ellipse-shaped split tubes denoted by 1 and 2, reproduced from [98]; (**d**) the single HR with olive neck, reproduced from [102]; (**e**) the sketch of sound absorptive panel with arrays of embedded coplanar spiral tubes, reproduced from [104].

Jimenez et al. presented theoretical and experimental evidence of subwavelength resonant panels, and it exhibits quasiperfect sound absorption at low frequencies, as shown in Figure 10a. The subwavelength panel is composed of periodic horizontal slits loaded by identical HRs. Due to the presence of the HRs, the propagation inside each slit is strongly dispersive, with near-zero phase velocity close to the resonance of the HRs. In this slow sound regime, the frequencies of the cavity modes inside the slit are down-shifted and the slit behaves as a subwavelength resonator. Furthermore, the strong dispersion causes cavity resonances below the HR resonance frequency, and quasi-critical coupling of symmetry and antisymmetry can be achieved simultaneously. So, quasiperfect absorption can be attained by using only monopolar resonators in a material that includes transmission [95].

A metasurface based on a subwavelength perfect sound absorber with coupled multiple resonators was designed and fabricated by Li et al which converts the incident wave to a non-radiating surface mode with matched impedance, thereby absorbing the incident energy and rendering it dark to the incident sound. Over 99% energy absorption is achieved in the experiment. The proposed metasurface yields near perfect absorption experimentally with subwavelength dimensions (λ/20) [96].

Guo et al. propose an ultrathin metasurface for low-frequency sound absorption, which is composed of HR-like resonators with an embedded spiral neck and a coiling-up backing cavity (see Figure 10b). The analytical, numerical and experimental results show that the proposed metasurface can achieve excellent absorption (absorption coefficient being 0.98) at 180 Hz with an extremely thin thickness of 13 mm (λ/145); a dual-band low-frequency absorber and a wide-band one are achieved by multiple units with different geometric parameters in parallel [97].

Furthermore, near perfect absorption is obtained by an acoustic absorber based on split tube resonators [98] (see Figure 10c), resonators with symmetrical or anti-symmetrical coherent perfect absorption (CPA) [99], resonators with asymmetric configurations [100], an HR array [101] and so on [98,99,100,101]. In addition, the geometric parameters of the HR unit play an important role in acoustic absorption (see Figure 10d) [102,103].

Besides the above two types of structure unit, the Fabry–Perot (F-P) resonator can also be used to design the metasurface. The F-P channel is so narrow that dissipation occurs due to air sticking and high absorption is realized, but the size of this channel needs a minimum of one-quarter wavelength. However, an ultrathin sound absorbing panel (λ/100) composed by bending and coiling-up quarter-wavelength sound damping tubes was reported by Cai et al., as shown in Figure 10e. Absorption efficiencies of the absorbing panel were in good agreement between theoretical analysis and experimental measurements [104].

Yang et al. reported an absorbing metasurface composed of square lattice, which consists of 16 FP channels; blue channels are coiled by three foldings, pink channels are coiled by two foldings, orange channels are coiled by one folding, and the green channels are straight. Near-perfect flat absorption starting at around 400 Hz can be achieved by this metasurface [105].

**Figure 11:**
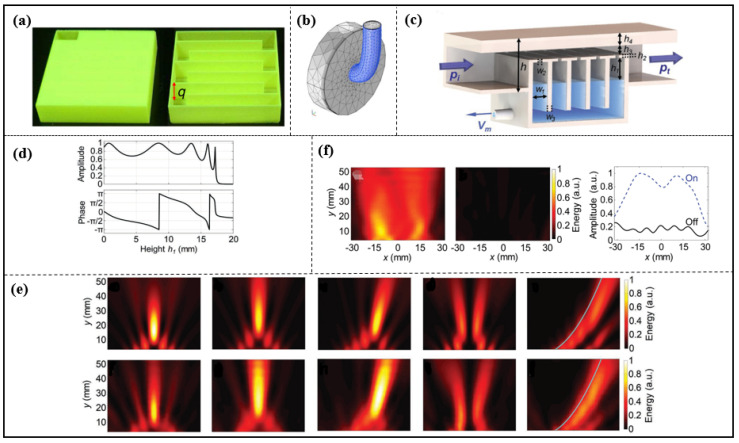
(**a**) Photographs of a realistic unit cell fabricated with polylacticacid (PLA) by means of (**left**) 3D printing and (**right**) its inner structure, reproduced from [107]; (**b**) bending quarter wavelength resonators, reproduced from [108]; (**c**) a schematic representation of a tunable subwavelength unit cell, which is composed of a straight channel and five shunted HRs. The cavity sizes are controlled by pumping fluid into/out of the unit cell, (**d**) numerical characterization of unit cell. By changing the cavity height of *h*_1_, the acoustic phase can be tuned in the full range of [−π, π] while maintaining high transmission amplitude. When the cavity height is over 18 mm, the transmission coefficient drops to zero, (**e**) analytical (**up**) and experimental (**down**) results for acoustic beam engineering. Analytical energy fields of five different acoustic beams, which demonstrate beamforming, tuning the focal distance, steering the beam direction, generating a tweezer-like beam and guiding energy along a parabolic trajectory, respectively, (**f**) experimental results for on/off switching of acoustic energy flow. Acoustic energy fields at (**left**) on and (**middle**) off states. (**Right**) comparison of pressure amplitudes along a line at *y* = 25 mm between on and off states. Reproduced from [82].

Moreover, the assembled structures with different length F-P channels [106], bending F-P channel array [107] (see Figure 11a), bending quarter-wavelength resonators [108] (see Figure 11b), and the unit composed of HRs and F-P channels [109] can also achieve absorption in different bands [106,107,108,109].

## 4. Tunable Metasurface

As mentioned above, acoustic metasurfaces have received extensive attention because of their multiple functionalities and ultrathin characteristics. However, most of the manufactured acoustic metasurfaces respond at a certain frequency. In other words, the working frequency region of the resonance metasurfaces is narrow and the function is single. The metasurface needs to be rebuilt if the working frequency or the desired functionality has a change, which will cause waste and limit its applications. So, it is necessary to design tunable acoustic metasurfaces, like the electromagnetic counterpart. In this section, we will give a brief review on the progress in tunable acoustic metasurfaces. Usually, there are two manners to achieve tunability. First, the mechanical reconfigurability of a unit itself is used to adjust the phase change in the gradient metasurface. Second, piezoelectric materials and magnetomechanical materials are employed to achieve reconfigurable elements.

### 4.1. Mechanical Reconfigurable Units

Tian et al. proposed a tunable metasurface, and its unit is composed of a straight channel and five shunted HRs (see Figure 11c). The phase and amplitude of transmission acoustic waves through each unit cell can be modulated dynamically and continuously (see Figure 11d), and its effective mass can be tuned by a robust fluidic system. Based on such a mechanism, the metasurface can achieve versatile wave manipulation by engineering the phase and amplitude of transmission waves on a subwavelength scale. Through acoustic field scanning experiments, multiple wave manipulation, including steering acoustic waves, engineering acoustic beams and switching on/off acoustic energy flow by using one design of a metasurface, is visually demonstrated (see Figure 11e,f) [82].

**Figure 12:**
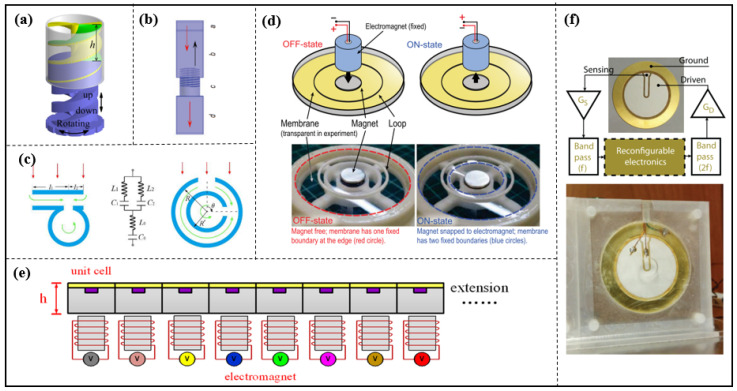
(**a**) The cylindrical unit of the tunable metasurface, reproduced from [110,111]; (**b**) schematic of helix, reproduced from [112]; (**c**) (**left**) schematic sketch and (**middle**) equivalent circuit of the coupled structure by split hollow sphere (SHS) and hollow tube (HT). (**Right**) schematic sketch of the optimized model based on (**left**); the inner ring can be spun freely to arbitrary angle θ around its center axis, reproduced from [118]; (**d**) the unit cell design. The membrane can be electrically switched between two states; one (denoted OFF state) has one fixed boundary at its edge and the other (denoted ON state) has two fixed boundaries, reproduced from [122]; (**e**) schematic diagram of the proposed magnetic-controlled metasurface, reproduced from [41]; (**f**) (**up**) unit cell consisting of a piezoelectric membrane. The cell acoustic response is controlled by a digital electronic circuit that can be reconfigured in real-time, (**down**) photograph of the fabricated unit cell. Reproduced from [124].

Fan et al. theoretically and experimentally investigated a helical acoustic metasurface capable of providing a modulated sound-reflected wavefront and a continuously tunable broadband feature, as shown in Figure 12a. The metasurface experimentally demonstrated the continuously tunable multifunction, including anomalous reflection, arbitrary focusing, self-bending beams, broadband carpet cloaking (curved metasurface) and ground illusion at a wide working band (curved metasurface) [110,111].

A flat, structurally tunable acoustic metasurface is constructed based on the helical unit (see Figure 12b). The length of the acoustic channel can be tuned by the screw-in depth of the helix, and then, the wave phase for the transmission acoustic wave can be tuned and the wavefront can be manipulated. Just by screwing in or out the helixes, multifunction, such as anomalous refraction, point focusing, beam focusing and self-bending, can be realized and switched, and the broadband operating frequency is also realized. The experiments for anomalous refraction and point focusing are also performed, and the results show that the designed metasurface is effective [112].

A reconfigurable acoustic metalens is realized by utilizing an existing active metasurface. By tuning the position of the sliders inside each unit cell with a dynamic control system, arbitrary scanning of the focus can be achieved. Its trajectory can also be flexibly manipulated under basic transformations including rotation, translation and scaling. These results have been confirmed with full wave simulations and measurements [113]. Tuning the slit width [114] of a composite unit with a HR array and tube or using the slider [115] to adjust the volume of the cavity can both achieve acoustic phenomena with efficient transmission such as sound wave redirection, focusing and acoustic illusion [114,115].

Additionally, the acoustic phenomena can be realized by varying the helical length, rotating the angle of resonator (see Figure 12c) and tuning the distance of the hard boundary, and these phenomena are anomalous reflection, perfect absorption, acoustic axicon for the Bessel beam or Airy beam, tunable carpet cloak, and indifferent bands, both airborne or waterborne-filed [32,116,117,118,119,120].

A metasurface was proposed by Li et al. in 2019 which is composed of a square lattice of circular holes with gradient annular bumps. The phase shift is tuned by changing the volume of water filled in the holes. The numerical results show that the acoustic focusing on a subwavelength scale is obtained by selecting a suitable water depth, and the wavefront of the reflected wave can be manipulated over a wide frequency range [62]. Tunable curved metasurfaces based on this unit cell with corrugated holes are designed, and anomalous reflection, focusing and ground illusion are numerically demonstrated [121].

### 4.2. Electromagnetic Reconfigurable Units

A membrane unit fixing an electromagnet was proposed by Ma et al. in 2018 (see Figure 12d), and tunability can be achieved by changing the current level and the magnitude force of the electromagnet and then adjusting the film tension [122]. A magnetically controlled approach was investigated for achieving a multifunctional acoustic metasurface with elastic films and additional mass. The properties of this acoustic metasurface could be continuously modulated by magnetic force value. Through switching the direction of the magnetic forces, the transmission acoustic wave is easily tailored, and different functions such as focusing, beam splitting-like and other near-field acoustic displays are switched [123].

As shown in Figure 12e, a magnetic-control multifunctional metasurface based on membrane structures with magnetic response at deep subwavelength scales (~λ/85) was proposed for low-frequency wave manipulation by Chen et al., and extraordinary phenomena, such as acoustic wave redirecting, focusing, bending, etc., were realized by switching the magnetic force distribution without changing the physical structure over a wide band [41]. A metasurface is composed of the piezoelectric membrane (see Figure 12f) and transducer, which can change its local acoustic response almost arbitrarily in real time. A metasurface with a variety of functions, such as lenses and beam steering, and the efficient second harmonic acoustic imaging that overcomes the diffraction limit of linear lenses was experimentally demonstrated [124].

## 5. Conclusions and Outlook

This paper reviews research progress on resonant metasurfaces, including the design of structural units, metasurface classification and tunability. Overall, the subject of metasurfaces remains challenging, with many unresolved issues from their design to application.

It is crucial for resonant metasurfaces to design structure units. Further, exploring structure units based on bioinspiration presents an intriguing topic [125]. Airborne acoustic metasurfaces have received much attention, but there is a noticeable lack of research on waterborne acoustic ones because the wavelength of sound in water is longer and the propagation loss is smaller. Therefore, controlling sound in water is more challenging than controlling sound in air of the same frequency. Furthermore, the density and impedance of water are larger than those of air, so conventional metal cannot be considered rigid and becomes an elastomer. Additionally, the fluid loading of water on the structure cannot be ignored, making the design of water acoustic metasurfaces more complex.

The study of tunable metasurfaces have been made greater progress; however, tuning a phase gradient metasurface is more difficult because of the required precise phase shift profile for a particular functionality. Usually, each element should be tuned independently, which makes tunability difficult to realize. Therefore, new mechanisms and methods of tunability need be explored. Using chips in structural units to design intelligent tunable structural units that can be tuned autonomously on demand is an interesting and challenging topic.

The design of broadband metasurfaces has been a widely concerned but difficult problem. As one of the possible solutions, the tunability design of a metasurface by adjusting the structural geometry or material properties of the unit could be considered in order to obtain the same response at different frequencies. However, in the case of broadband pulse incidence, this approach is no longer applicable; reverse-design topology optimization provides a possible solution [126]. In addition, due to the complexity of realistic acoustic fields, the use of multiple coupled structural units to realize simultaneous acoustic modulation in multiple frequency bands is also an interesting solution [61].

Customized metasurfaces for specific functions are appealing for practical applications, yet challenging to achieve through experiential design. The active design of highly efficient broadband metasurfaces through resonant structure units with double-negative properties is also an interesting and challenging topic.

## Data Availability

Not applicable.
